# Optimization and characterization of antileukemic l-asparaginase produced by *Fusarium solani* endophyte

**DOI:** 10.1186/s13568-023-01602-2

**Published:** 2023-09-13

**Authors:** Sarah Osama, Moshera M. El-Sherei, Dalia A. Al-Mahdy, Mokhtar Bishr, Osama Salama, Marwa M. Raafat

**Affiliations:** 1 Pharmacognosy and Medicinal Plants Department, Faculty of Pharmacy, Future University in Egypt, Cairo, Egypt; 2 Department of Pharmacognosy, Faculty of Pharmacy, Cairo University, Cairo, 11562 Egypt; 3 Arab Company for Pharmaceuticals and Medicinal Plants (Mepaco), Cairo, Egypt; 4 Microbiology and Immunology Department, Faculty of Pharmacy, Future University in Egypt, Cairo, 11835 Egypt

**Keywords:** l-asparaginase, Endophytes, *Fusarium solani*, *Hedera helix*, Optimization, Characterization, Cytotoxicity

## Abstract

l-asparaginase is an antileukemic enzyme that hydrolyzes l-asparagine into l-aspartic acid and ammonia, causing cell starvation and apoptosis in susceptible leukemic cell populations. Currently, l-asparaginase obtained from bacterial sources is constrained by several issues, including lesser productivity, stability, selectivity, and higher toxicity. The goal of this study is to provide fungal l-asparaginase with *in-vitro* effectiveness towards different human carcinomas. l-asparaginase from endophytic *Fusarium solani* (Gene Bank accession number MW209717) isolated from the roots of the medicinal plant *Hedera helix* L. was characterized and optimized experimentally for maximum l-asparaginase production in addition to evaluating its subsequent cytotoxicity towards acute monocytic leukemia and human skin fibroblast cell lines. The enzyme production was maximized using potato dextrose media (15.44 IU/ml/hr) at the 5th and 6th days of fermentation with incubation temperature 30 °C, 3% asparagine, 150–180 rpm agitation rate and a 250 ml flask. Enzyme characterization studies revealed that the enzyme maintained its thermal stability with temperatures up to 60 °C. However, its optimal activity was achieved at 35 °C. On measuring the enzymatic activity at various temperatures and different pH, maximum enzyme activity was recorded at 40 °C and pH 8 using 0.1 M asparagine concentration. Results also revealed promising cytotoxic activity against acute monocytic leukemia with IC_50_ = 3.66 µg/ml and low cytotoxicity against tested normal human skin fibroblast cell line which suggested that it might have selective toxicity, and consequently it could be used as a less toxic alternative to the current formulations.

## Introduction

Acute lymphoblastic leukemia (ALL) is a type of white blood cells (WBCs) cancer. It is distinguished by the increased development of malignant and immature WBCs (lymphoblast) in the bone marrow. Chemotherapy, steroids, radiation therapy, and enzyme therapy are some of the current treatment options (Onciu 2009).

**Fig. 1:**
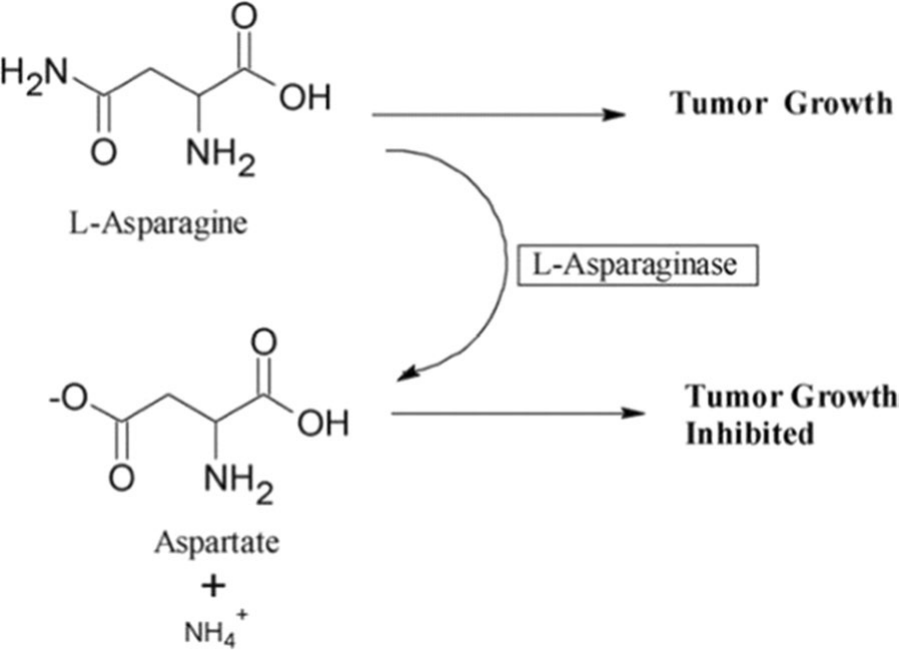
Schematic representation of l-asparaginase enzyme mechanism of action (Narta et al. 2007)

Enzyme therapy aims to deplete a certain amino acid which is considered essential for tumor cell growth but not needed by normal tissues. This treatment strategy is regarded as a novel and encouraging method to the challenge of treatment of leukemia and disseminated cancer. Among the microorganisms-derived enzymatic drugs used is the l-asparginase (l-ASNase) enzyme (Pieters et al. 2011).

l-ASNase is commonly used in cancer therapy in both pediatric and adult procedures. It is regarded as the foundation for acute lymphoblastic leukemia treatment. It plays a critical part in storage and transport of nitrogen which is needed for biosynthesis of protein. Malignant cells synthesise it at a slower rate than normal cells owing to their reduced capability to synthesise l-asparagine synthetase (Abdelrazek et al. 2020). l-ASNase hydrolyzes l-asparagine to aspartic acid and ammonia. As many kinds of tumour cells require l-asparagine for protein synthesis, the presence of l-ASNase deprives them of a critical growth factor, resulting in cytotoxicity against leukemic cells, rendering them incapable of synthesising protein, RNA, and DNA, as well as inducing apoptosis in these cells Fig. 1 (Jain et al. 2012).

This enzyme is found in many bacteria, and fungi including: *Aerobacter*, *Bacillus*, *Pseudomonas*, *Serratia*, *Xanthomonas*, *Photobacterium*, *Streptomyces*, *Proteus*, *Vibrio* and *Aspergillus* species as well as plants, however, it’s not found in humans.The physiochemical properties as well as the kinetic parameters of the enzyme differ depending on the source. Nowadays, it is produced commercially from two major bacterial sources: recombinant *Escherichia coli* and *Erwinia chrysanthemi* (Muneer et al. 2020). However, because of their toxicity, which includes thrombosis, pancreatitis, hyperglycemia, and hepatotoxicity, the use of bacterial l-ASNases for pharmacological uses raises concerns. Furthermore, malignant cells are resistant to l-ASNase preparations of bacterial origin, even yet posing another significant challenge in cancer treatment (Baruchel et al. 2020). Alternatively, according to several studies, fungal l-ASNase was proven to be lacking any unfavorable conditions. l-ASNase produced by *Aspergillus*, *Penicillium*, *Fusarium*, and other fungal species showed no adverse effects (EL-Gendy et al. 2018, 2021; Benchamin et al. 2019a, 2019b; Prihanto et al. 2019).

These findings drew the attention of researchers to hunt for and locate new more selective and less toxic alternate l-ASNase sources (Muneer et al. 2020), and endophytes were identified as one of the microbial groups of interest.

Endophytes are abundant organisms living in plants either inter-cellularly or intra-cellularly for at least part of their lives without causing harmful effects to the host. They are found in nearly all plants and deemed to be a novel source which can be explored to alleviate many issues faced by humankind. They are considered to be a chemical reservoir for many novel compounds, including anticancer, antiviral, immunomodulatory, antioxidant, and anti-inflammatory compounds (Schulz et al. 2002; Guo et al. 2008; Kodoli et al. 2021).

Ever since the endophytic fungus *Taxomyces andreanae* yielded the multimillion-dollar medicine Taxol, endophytic fungi have been investigated as possible sources of anticancer drugs (Kaul et al. 2012).

The present study aimed at screening of l-ASNase producing endophytic fungi isolated from *Hedera helix* L. plant as an alternate source for bacteria with greater productivity, stability, and efficacy, followed by experimental tactics for the characterization, and optimization of the enzyme production. Additionally, the enzyme antileukemic activity was assessed.

## Materials and methods

### Plant material

*Hedera helix* L. plant was obtained from the Arab Company of Pharmaceuticals and Medicinal Plants (Mepaco-Medifood) EL-Sharkya, Egypt with coordinates near (30.3799◦ N, 31.4544◦ E). It was reserved at room temperature in well ventilated place. Dr. Mokhtar Bishr, Technical Director of Mepaco Company, confirmed the identity of the plant samples.

### Isolation of endophytic fungi

*Hedera helix* L. endophytic fungi were isolated by the technique mentioned formerly by (Hazalin et al. 2009) with some modifications. In brief, the different plant parts including leaves, stem and roots were washed several times with running water and dried. They were sequentially dipped in 70% ethanol three times each for 1 min., followed by five times rinsing with sterile water after each time of sterilization. Dried sterilized plant parts were cut into 1 mm segments and aseptically inoculated on Potato Dextrose Agar (PDA) (Himedia, Mumbai, India) plates which were supplemented with 25 µg/ml of both streptomycin and gentamicin to prevent bacterial growth. As negative controls, non-inoculated PDA plates were used, as well as PDA plates inoculated with 1 ml of last step washing water. All plates were incubated at 25 °C for seven to fourteen days. Different mycelia emerging out of the segments were cultivated, isolated, and the isolated pure fungi were preserved on PDA slants at 4 ℃.

### Screening of l-ASNase-producing endophytes

The ability to produce l-ASNase was tested in all isolated endophytic fungi. The qualitative detection assay was performed in accordance with the method published by (Izadpanah et al. 2014). The assay depends on the ability of l-asparaginase producing isolates to split L-asparagine with production of ammonia leading to the formation of a pink zone in presence of phenol red indicator. Sterile modified M9 agar medium (pH 7·0) (g/l): Na_2_HPO_4_, 6·78 g; KH_2_PO_4_, 3·0 g; NaCl, 0·5 g; NH_4_Cl, 1 g; 1M MgSO_4_.7H_2_O, 2·0 ml; 0·1M CaCl_2_.2H_2_O, 1·0 ml; 20% glucose stock, 10.0 ml; agar 20·0 g.) containing 1% w/v asparagine and phenol red were inoculated with the isolated endophytic fungi. All plates were incubated for 5 days at 25 ℃. Pinc zones surounding the colonies indicated l-ASNase producing active isolate.

### Molecular identification of l-ASNase-producing endophytic fungi

To amplify the ribosomal Internal Transcribed Spacer (ITS) region, genomic DNA was extracted by Sigma Scientific Services Co. ITS 1 (5′-TCC GTA GGT GAA CCT GCG G-3′) and ITS 4 (5′-TCC TCC GCT TAT TGA TAT GC-3′) were used as forward and reverse primers, respectively. The following Polymerase Chain Reaction (PCR) thermal cycling parameters were used: initial denaturation at 95°C for 10 min, 35 cycles of denaturation at 95 °C for 30 s, annealing at 57 °C for 1 min, and extension at 72 °C for 1.5 min. The post cycling expansion was completed in a single cycle at 72 °C for 10 min. The PCR products were then purified using GeneJET PCR Purification Kit (Thermo K0701, Waltham, MA, USA) according to the company’s instructions and the refined DNA was stored at − 20 °C. As a final step, the ABI 3730xl DNA sequencer was used to sequence the refined PCR product. Using Using National Center for Biotechnology Information (NCBI) BLAST (Basic Local Alignment Search Tool; http://blast.ncbi.nlm.nih.gov/), the isolated endophytic fungi’s final sequence was matched against sequences present in the GenBank database. The identified isolate sequence was deposited to GenBank database and assigned an accession number (Simpson et al. 2000; Morgan et al. 2009).

### l-ASNase quantitative assay

A quantitative assay of L-ASNase production was performed in all l-ASNase producing isolates in order to select the isolate with higer productivity. l-ASNase activity was estimated following the technique mentioned by (Mashburn and Wriston 1963). Fungal isolates (2% v/v from adjusted growth at OD600nm = 1.0) were inoculated in 250 ml Erlenmeyer flasks containing 50 ml modified M9 broth supplemented with 1% w/v asparagine at 25 °C and 180 rpm for 5 days. Extracellular l-ASNase were obtained in the supernatant after cooling centrifugation at 1957 × *g* and 4 °C for 20 min (Jain et al. 2012). The obtained crude L-ASNase extract was estimated by the nesslerization method (Mashburn and Wriston 1963; Mahajan et al. 2014) which depends on the ability of the enzyme preparation to hydrolyze l-asparagine to release ammonia. The reaction mixture contained 0.5 ml of enzyme sample, 0.5 ml 0.05M Tris–HCl buffer (pH 8.6) and 0.5 ml 0.04M asparagine. It was incubated at 37 °C for 30 min. The enzyme activity was stopped by adding 10% w/v Tri-chloroacetic acid (TCA). The mixture was centrifuged at 10,000 rpm for 5 min then 3.7 ml of distilled water was added to 0.1 ml of the supernatant followed by 0.2 ml Nessler's reagent and kept for 20 min at room temperature. The absorbance was measured at 480 nm using a spectrophotometer. The amount of ammonia liberated was calculated using ammonium sulphate standard curve (Jain et al. 2012). At 37 °C and pH 8.6, one unit of l-ASNase activity equals the amount of enzyme required to release one µmol of ammonia per hour (Mahajan et al. 2014; Abdelrazek et al. 2019).

Endophytic fungus with the highest l-ASNase production ability was selected for further studies to characterize the enzyme and achieve optimum conditions required for enzyme production.

### Characterization of l-ASNase enzyme

The crude l-ASNase preparation was examined for different characteristics to determine its application range. These included temperature stability, optimal temperature, and activity with various asparagine concentrations (substrate). All assays were done in triplicates.

### l-ASNase thermal stability

The crude enzyme preparation was incubated at temperature range from 25 to 60 °C for 30 min then cooled in an ice bath immediately. The residual enzyme activity was detected by nesslerization method as previously described. The residual enzyme activity percentage was calculated relative to the untreated enzyme preparation.

### l-ASNase activity at different temperatures

This was performed by conducting the nesslerization assay at different reaction temperatures ranging from 25 to 45°C then the enzyme activity was compared to that obtained at 37 °C.

### l-ASNase activity at different pH values

This was carried out by determination of the enzyme activity at different reaction pH values (4, 5, 6, 7 and 9) using 0.05M Tris HCl buffer for pH 6, 7 and 9 and 0.05M Phosphate buffer for pH 4 and 5. The activity was then compared with that obtained at original pH 8.6.

### Substrate saturation concentration

A range of 0.02–0.14 M l-asparagine concentrations were included in the nesslerization assay. l-ASNase activity determined was compared to that detected at 0.04 M l-asparagine concentration.

### Optimization of l-ASNase production (one factor at a time)

Each of the following parameters were tested individually using the selected isolate with highest l-ASNase production. At the end of each test and at the end of incubation, l-ASNase activity was quantitatively determined as previously mentioned. All assays were carried out in triplicates.

### Effect of different culture media

Three different media were used; M9 broth, rice medium, and Potato Dextrose Broth (BDB, (Himedia, Mumbai, India) as minimal microbial medium, solid medium, and enriched liquid medium, respectively. For the preparation of solid rice medium, 10 g of rice were mixed with 25 mL of deionized H_2_O in a 250 mL Erlenmeyer flask, sealed with a cotton plug, and autoclaved.

All media were supplemented with 1% w/v asparagine, inoculated with adjusted fungal inoculum of 1 OD600, and incubated at 25 ℃ for 5 days.

### Effect of incubation temperature

The quantitative assay was carried out as described before, with the flasks inoculated with the adjusted isolate were incubated at different temperatures (20, 25, 30, 37, and 45 °C).

### Effect of incubation time

The assay was carried out as mentioned previously except that the incubation time of the inoculated flasks was different (4,5, 6, and 7 days).

### Effect of agitation

Different levels of agitation (100, 150, 180, 200, and 250 rpm) were tested for their effect on L-ASNase activity.

### Effect of aeration

The assay was performed using different Erlenmeyer flasks capacities (100, 250, 500, and 1000 ml) containing 50 ml PDB supplemented with 1% w/v asparagine and inoculated with 2% v/v adjusted fungal isolate.

### Determination of l-ASNase anti-leukemic activity by WST-1 assay

Acute monocytic leukemia (THP1) and Human Skin Fibroblast (HSF) cell lines were provided by Nawah Scientific Inc. (Mokattam, Cairo, Egypt)**.** THP1 cells were reserved in Roswell Park Memorial Institute (RPMI) medium (Thermo Fisher Scientific Inc., United States), while HSF cells were kept in Dulbecco’s Modified Eagle Medium (DMEM, Thermo Fisher Scientific Inc., United States). Both media were supplemented with 100 mg/mL streptomycin, 100 units/mL penicillin, and 10% heat-inactivated fetal bovine serum, and incubated in a humidified 5% (v/v) CO_2_ atmosphere at 37 °C (Sharma et al. 2014; Alaufi et al. 2017).

The anti-leukemic activity of crude L-ASNase enzyme at different concentrations (ranging from 0.01 and 300 μg/ mL) was estimated against THP1 cell line. HSF cell line was also used to estimate the cytotoxic activity of L-ASNase on normal cells. Cell viability was assessed by water-soluble tetrazolium-1 (WST-1) assay using Abcam® kit (ab155902 WST-1 Cell Proliferation Reagent). Using 96-well plates, aliquots of 50 µL cell suspension (3 × 10^3^ cells) were seeded and incubated in complete media for 24 h. Cells were given additional aliquot of 50 µL media containing enzyme at different concentrations. Cells were treated with 10 µL WST-1 reagent after 48 h of enzyme-cell contact, and absorbance was measured at 450 nm after 1 h using a BMG LABTECH^®^- FLUOstar Omega microplate reader (Allmendgrün, Ortenberg). The amount of enzyme inhibiting 50% viability was calculated (IC_50_). Results were expressed as a viability percentage versus untreated control cells (100% viability). For each condition, triplicate wells were tested, and standard deviations were calculated (Sharma et al. 2014; Alaufi et al. 2017).

### Statistical analyses and graphical representations

All experiments were done in triplicates. The outcomes of each experiment were expressed as an average with a standard deviation. Both the mean and standard deviation (shown as error bars) were calculated. Graph Pad Prism Version 5.0 software was used for all data analysis.

## Results

### Isolation and identification of l-ASNase producing endophytic fungi

**Fig. 2:**
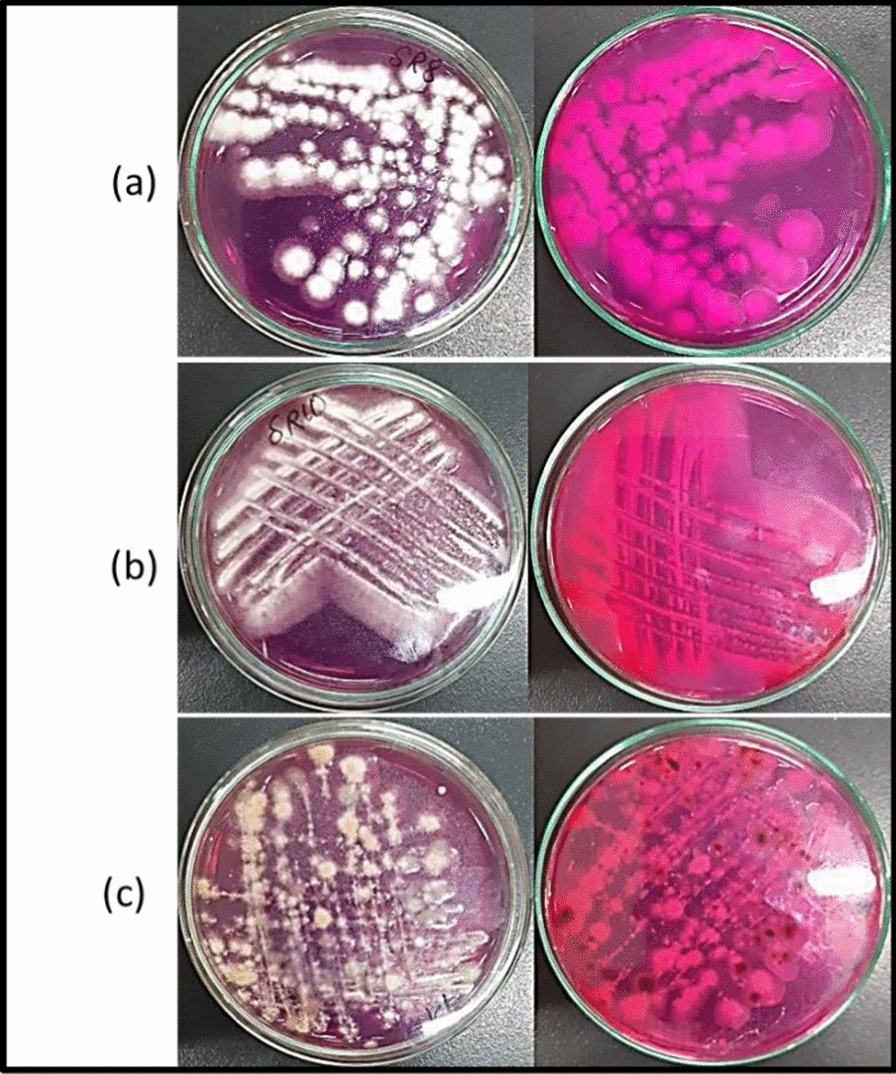
Qualitative l-ASNase activity. l-ASNase activity indicated by pink color on modified M9 agar plates. The 3 L-ASNase producing fungi (**a**) *Talaromyces trachyspermus* (**b**) *Fusarium solani* (**c**) *Saccharomycopsis fibuligera*

Eight endophytic fungi were isolated from *Hedera helix* L. All of them were investigated qualitatively for their l-asparaginase production ability. The isolates were inoculated on modified M9 agar plates containg 1% l-asparagine and phenol red as indicator. l-ASNase producing isolates hydrolyzed l-asparagine to aspartic acid and ammonia, increasing the pH of the medium indicated by conversion of phenol red color to pink. Three fungal isolates showed a greater intensity of pink coloration Fig. 2. l-ASNase producing isolates were molecularly identified using ribosomal ITS region and submitted to GeneBank database with accession numbers. The identified isolates were *Talaromyces trachyspermus* MW131876, *Fusarium solani* MW209717 and *Saccharomycopsis fibuligera* MW165540.

**Fig. 3:**
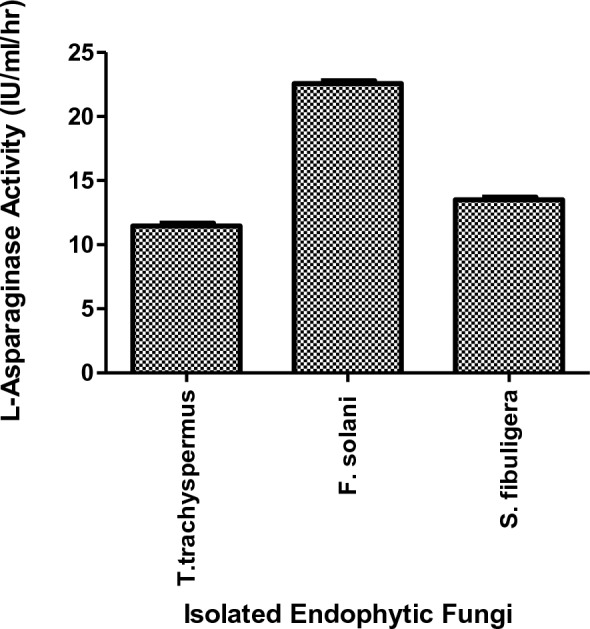
Quantitative l-ASNase activity. *Fusarium solani* showed higher L-ASNase activity, followed by *Saccharomycopsis fibuligera* then* Talaromyces trachyspermus*

The three isolates were quantitatively tested for enzyme activity and the results revealed that *Fusarium solani* showed highest l-ASNase production (22.76 IU/ml/hr) relative to the other two isolates *Talaromyces trachyspermus* and *Saccharomycopsis fibuligera* (11.58 IU/ml/hr and 13.54 IU/ml/hr, respectively), accordingly *Fusarium solani* was selected for additional characterization and optimization studies on L-ASNase production Fig. 3. *Fusarium solani* MW209717 strain has been deposited at the Culture collection of Cairo "MIRCIN" under numbers EMCC 28560, Agric. Faculty, Ain Shams University).

### Characterization of the l-ASNase produced by *Fusarium solani* isolate

**Fig. 4:**
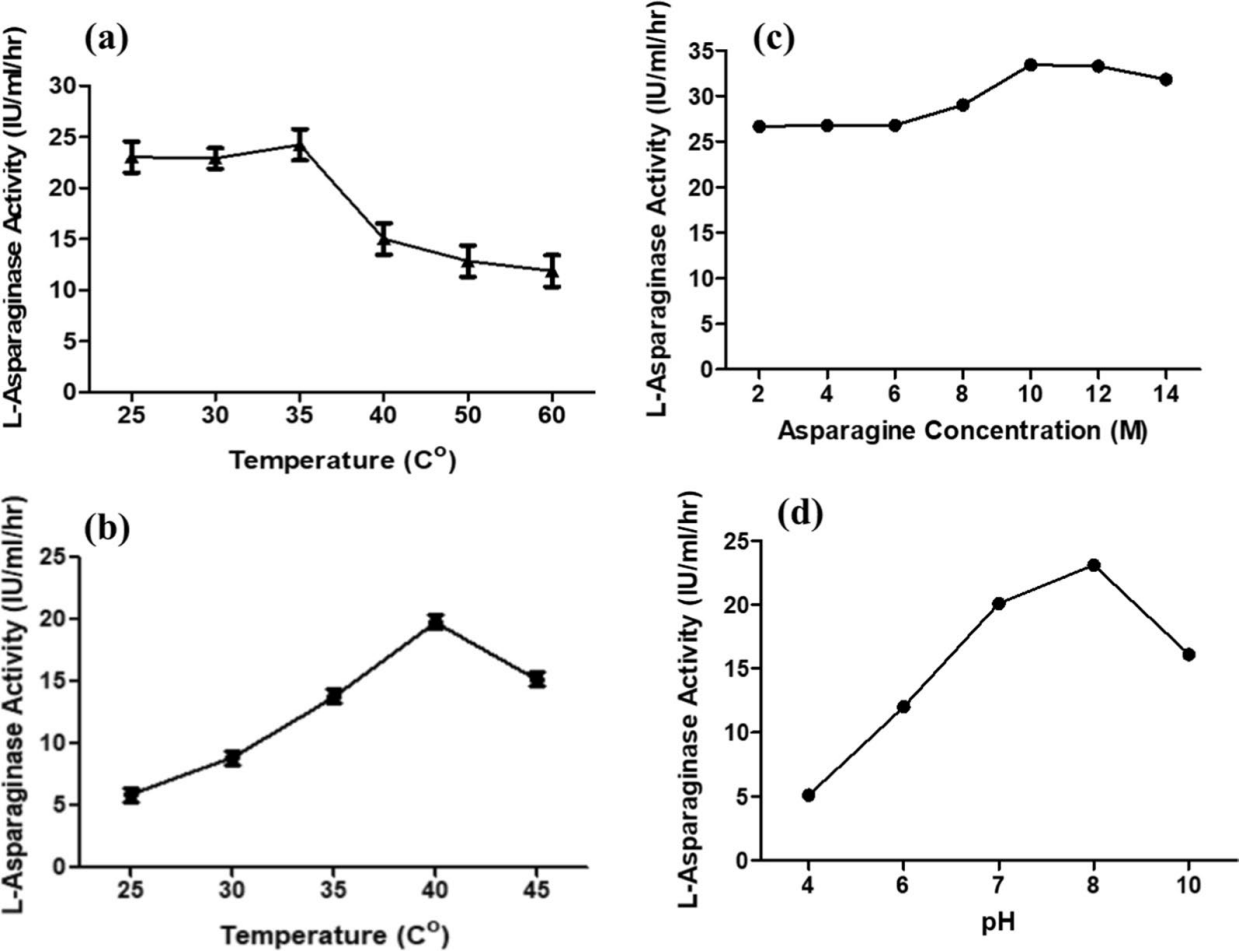
l-Asparaginase Characterization: **a** Thermal Stability, **b** Activity at various temperatures, **c** Different L-asparagine concentrations, **d** Activity at various pH values

The crude enzyme preparation was characterized for some industrial parameters which included thermal stability, optimum temperature, pH, and substrate concentration required for l-ASNase activity. Thermal stability studies revealed that the tested isolate’s l-ASNase was stable at a wide range of temperatures, and it could withstand temperature treatments up to 60 °C for 30 min without significantly reducing its activity. Whereas it’s optimal activity was achieved at 35 °C. Also, on measuring the activity of the enzyme at various temperatures, results indicated that the l-ASNase activity was not significantly affected when subjected to temperature up to 45 °C for 30 min. The highest activity was detected at 40 °C. Regarding the pH, the results showed that l-ASNase activity increased gradually by increasing the pH reaching its maximum activity at pH 8. No enzyme activities were detected at pH lower than 4. To determine the saturation concentration of l-asparagine substrate, different l-asparagine concentrations were used ranging from 0.01 to 0.14 M. It was found that l-ASNase activity increased steadily by increasing the l-asparagine concentration showing the highest activity (33.421 IU/ml/hr) using 0.1 M asparagine concentration Fig. 4.

### Optimization of l-ASNase production by *Fusarium solani*

Different environmental conditions were applied for optimization of enzyme production including: different media, incubation time, incubation temperature, agitation, aeration, and different concentrations of asparagine.

To acquire higher growth and production of the enzyme, a comparative analysis was done by inoculating an adjusted inoculum of *Fusarium solani* in different culture medium. Modified M9 medium supporting the microorganism with the minimal requirements for growth, PDB enriched medium containing potato extract and dextrose in addition to rice media as solid-state fermentation medium. All media were supplemented with fixed concentration of l-asparagine and incubated under the same conditions without agitation.

The results showed that the highest l-ASNase production was obtained in PDB (15.44 IU/ml/hr) followed by modified M9 medium (8.45 IU/ml/hr) while solid rice medium showed very low productivity. Accordingly, all subsequent studies were conducted in PDB.

**Fig. 5:**
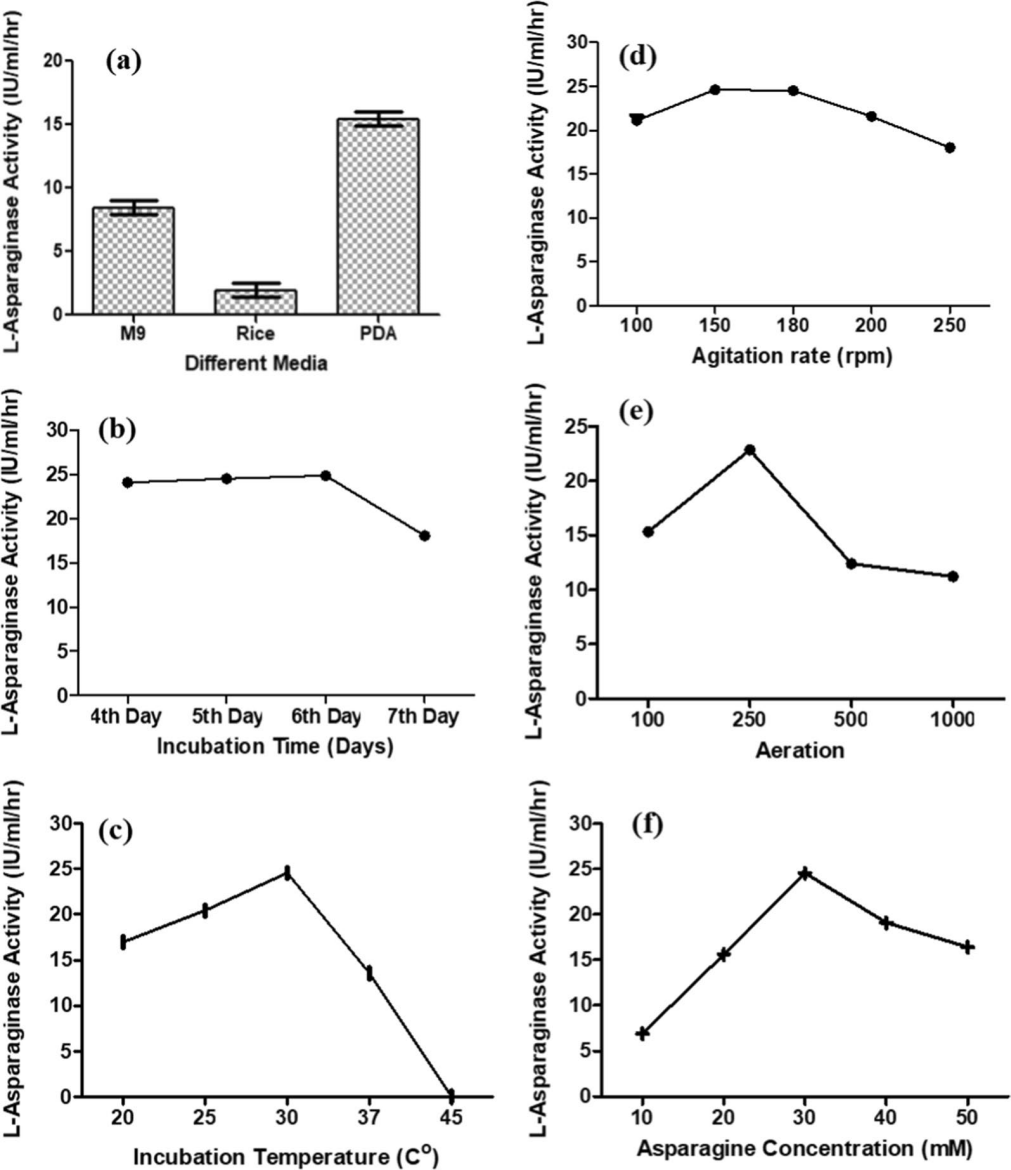
l-ASNase optimization factors: **a** Different Media, **b** Incubation Time, **c** Incubation Temperature, **d** Agitation Rate, **e** Aeration, **f** Asparagine Concentration

Incubation temperature and time are crucial parameters for microbial growth and production of enzymes and metabolites. Regarding the effect of incubation time, results indicated a gradual increase in the l-ASNase production reaching its maximum activity on the 5th and 6th days followed by a sharp decrease on the day seven. The assay of incubation temperature effect showed an escalation in the levels of l-ASNase production starting at 20 °C, reaching its maximum level at 30 °C and slightly declining thereafter at 37 °C until it almost completely disappeared at 45 °C Fig. 5.

Upon investigating the effect of agitation rate, the highest l-ASNase productivity was achieved at the range 150–180 rpm, with fewer productivities obtained at agitation rates out of this range. To test the effect of aeration using Erlenmeyer flasks with different volumes (100, 250, 500, and 1000 ml). Flasks with moderate aeration (250 ml flask) were optimum for the highest l-ASNase production. Concerning the effect of different asparagine concentrations on the l-ASNase production, the best activity was observed with 3% asparagine Fig. 5.

### Anti-leukemic activity of *Fusarium solani’s*L-ASNase

**Fig. 6:**
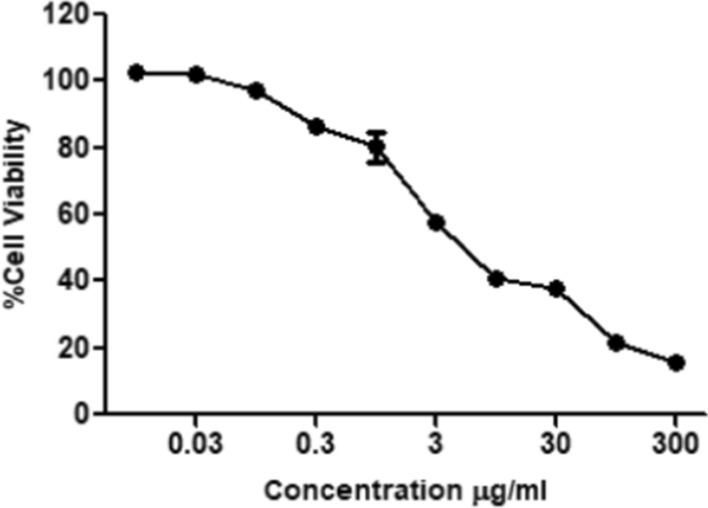
Cell viability of acute monocytic leukemia (THP1) after the treatment with different concentrations of the l-ASNase produced from *Fusarium solani* as measured by WST-1 Assay

**Fig. 7:**
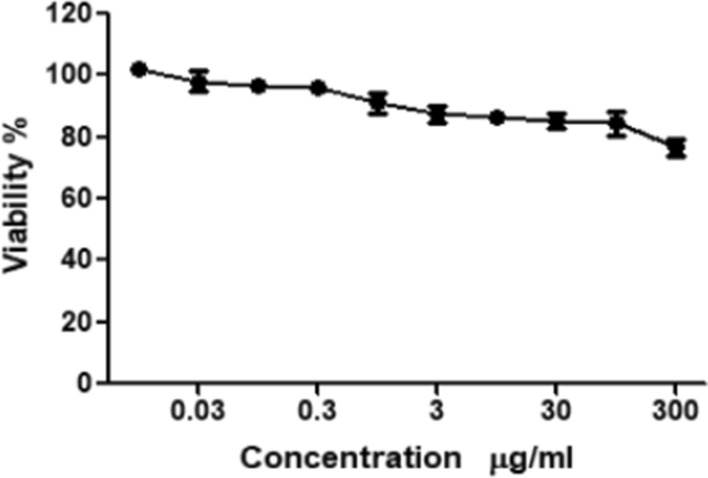
Cell viability of normal HSF cells after the treatment with different concentrations of the l-asparaginase produced from *Fusarium solani* as measured by WST-1 Assay

From the previous results, optimum conditions were selected and applied for antileukemic activity of l-ASNase produced by *Fusarium solani* on acute monocytic leukemia (THP1). Furthermore, the safety pattern of fungal l-ASNase was examined on normal human fibroblast cells. Both cell lines were treated with various increasing concentrations of l-ASNase ranging from 0.01 to 300 µg/mL Results revealed a dose dependent activity against the monocytic leukemia cell line with IC_50_ of 3.66 µg/mL Fig. 6. Low enzyme concentrations (0.01–10 µg/mL) revealed relative cell viability of almost 97–40.9% while high enzyme concentrations (30–300 µg/mL) revealed cell viability ranging from 37 to 15.6%. Surprisingly, l-ASNase showed mild toxicity towards normal human skin fibroblast cells (IC_50_ = ˃ 300 µg/mL), which suggests that it has selective toxicity Fig. 7.

## Discussion

l-ASNase is a chemotherapeutic agent that is used to treat a range of lymphoproliferative disorders and lymphomas, including ALL. For nearly 30 years, it has been a backbone of combination chemotherapy protocols used in the treatment of pediatric ALL (Narta et al. 2007). Because they are simpler to cultivate and easier to extract and purify l-asparaginase from, microbes are thought to be a better source of the enzyme, allowing for large-scale production (Asthana and Azmi 2003). For pharmaceutical purposes, l-ASNase is acquired from *Escherichia coli* and *Erwinia carotovora* (Cachumba et al. 2016). Nonetheless, allergic reactions could be triggered on by the bacterial source of this enzyme (Qeshmi et al. 2018).

The Joint FAO/WHO Expert Committee on Food Additives (JECFA) has declared that fungal l-ASNases are considered safe, this has been the case since 2007. However, the amount of l-ASNase produced does not match demand, thus new methods to boost yield, like the use of statistical tools, must be developed (Da Cunha et al. 2019).

In previous studies, several *Fusarium* spp. obtained from either plants, soil and marine algae were found to produce l-ASNase (Manasa and Nalini 2014). In fact, in a study examining 84 different fungal endophytes for their l-ASNase activity, an isolate of *Fusarium proliferatum* was observed to possess the best l-ASNase enzyme activity (Balbool and AbdeL-Azeem 2020; Hatamzadeh et al. 2020). Previous research investigated the L-ASNase produced by *Fusarium solani* along with the purification and characterization of the enzyme to obtain maximum yield (Uzma et al. 2016; Zaki et al. 2018; As-Suhbani and Bhosale 2020). The characterization and optimization of production conditions were proved in many studies to possess significant impact on the enzyme production (EL-Naggar et al. 2016; Zaki et al. 2018; EL-Gendy et al. 2021). In this study, experimental l-ASNase activity was analyzed to investigate the effect of various factors including: the use of different media, incubation period, incubation temperature, the effect of using different agitation rates, aeration as well as different asparagine concentration on production of l-ASNase by *Fusarium solani* isolated from the roots of *Hedera helix* L. plant.

Since enzymes have an inherent fragile nature, the difference between their native and denatured structures limit their applicability. In the industrial field, enzymes that are stable throughout a wide range of temperatures are in great demand. Thus, our goal was not only to increase l-ASNase production, but also to select l-ASNase with industrially required features.

One of the main parameters affecting the use of the enzyme in the pharmaceutical industry is its thermal stability as higher thermal stability is typically needed for industrial enzymes. Our findings showed that the enzyme had maximum stability at 35 °C with a 105.6% increase in activity compared to the control, and it maintained up to 61% of its activity at 40 °C. Additionally, the activity of the enzyme preparation from *Fusarium solani* increased gradually over temperatures ranging from 25 to 40 °C showing maximum activity at 40 °C (19.831 IU/ml/hr) with approximately a 338.9% increase in activity compared to the control (5.853 IU/ml/hr). However, the activity began to decline gradually at 45 °C where it achieved 76%. These findings came in agreement with the previously published study on *Bacillus licheniformis* where the activity of the test isolate’s enzyme preparation increased gradually from 20 to 35 °C and then decreased by approximately 27.7% at 45 °C. Its activity peaked at 40 °C (Abdelrazek et al. 2019). Similar findings were obtained with *Streptomyces gulbargensis* where the enzyme was observed to be maximally active at 40 °C (Amena et al. 2010).

Almost all isolated and purified l-ASNase from microbial sources were reported to operate in a basic medium with pH ranging from 8 to 10, whereas, any further increase in the alkalinity of the medium will lead to a dramatic decrease in enzyme activity (EL-Hefnawy et al. 2015; Al Yousef 2022). The optimum alkaline pH of the enzyme is attributed to the fact that the aspartate liberated by asparagine hydrolysis has lower affinity to the active catalytic site of the enzyme. This allows more asparagine to bind to the enzyme. At acidic pH, however, the enzyme's breakdown of asparagine results in the production of aspartic acid, which has a high affinity for the enzyme’s catalytic site, preventing asparagine from binding to the enzyme (Abdelrazek et al. 2019). These reported data confirmed our results where the best enzymatic activity was achieved at alkaline pH (pH 8) and any further increase in the alkalinity of the medium caused a decline in the enzyme activity. Similar results were obtained in *Aspergillus fumigatus* (Benchamin et al. 2019b), *Phaseolus vulgaris* (Mohamed et al. 2015), while in other *Fusarium* sp. the maximum activity was achieved at pH 9 (Asha and Pallavi 2012).

The highest activity of l-ASNase was found at 0.1M l-asparagine (33.42 IU/ml/hr) with nearly 125% increase in activity compared to the control (26.66 IU/ml/hr). and the activity decreased slightly with increasing substrate concentrations which might be explained by the substrate saturating the active enzyme sites. Similar findings were reported by (Kumar and Janakiraman 2015) in *Fusarium* species.

To obtain better growth and enzyme production, the culture was inoculated in several media for comparative examination. According to the findings, PDB led to better enzyme activity compared to the other media used. PDB was therefore selected as the media of choice for all upcoming experiments.

On analyzing the results obtained from studying the optimum incubation time for asparaginase production, differences in enzyme level produced was observed with different incubation periods. Quantitative analysis of the enzyme showed that its peak activity was achieved on the 6th day of incubation. Incubation beyond that time period results in a decline in enzyme production, which might be caused by the inactivation of the enzyme due to the presence of proteolytic activity or by the organism’s growth reaching a point where it is no longer able to maintain a stable growth in balance with the availability of nutrient resources. The enzyme activity declined with extended incubation times, which may be related to nutritional depletion or accumulation of hazardous end products (Varalakshmi and Raju 2013).

Short incubation periods are generally preferred for commercial enzyme manufacture as they are more economical and reduce the decomposition liability of the produced enzyme (Abdelrazek et al. 2019). Similar results were obtained from a study conducted on *Fusarium solani*, where five days was the ideal incubation period (Isaac and Abu-Tahon 2016). Microorganisms produced l-ASNase at different maximum incubation times, as revealed by studies on *Aspergillus terreus* and *Aspergillus niger* (Varalakshmi and Raju 2013) which indicated that incubation beyond 96 h showed a decline in enzyme production, 48 h for *Emericella nidulans* (Jayaramu et al. 2010) and 120 h for *Fusarium* spp. (Murali 2011).

Studying the effect of incubation temperature is considered one of the most significant factors affecting the enzyme productivity for maximum yield. The significance of the incubation temperature is due to the fact that it could determine the effects of inhibition, cell viability and death. Because it controls microbial proliferation and subsequently enzyme secretion, it is regarded as a crucial environmental element for l-ASNase synthesis by microbes. The incubation temperature assay was done at different temperatures viz*.,* 20, 25, 30, 35, 37 and 45 °C to examine their effect on enzyme production. Results revealed that the incubation temperature had a major impact on the production of l-ASNase with the maximum enzyme yield occurring at 30 °C.

Higher temperatures showed a decline in enzyme production until it completely stopped at 45 °C. This could be attributed to the deceleration in the microorganism’s metabolic activity as previously mentioned by (EL-Hefnawy et al. 2015). Similar findings were obtained from a study on *Fusarium solani* where maximum enzyme productivity was obtained at 30 °C (Isaac and Abu-Tahon 2016). Another study examining the enzyme obtained from *Fusarium* sp. also reported the optimum temperature to be between 30 and 40 °C for l-ASNase activity, whereas higher temperatures resulted in a reduction in enzyme activity, and the activity completely vanished at a temperature from 50 to 60 °C (Al Yousef 2022). Our results were also in agreement with other studies done on various microorganisms, where (Varalakshmi and Raju 2013) proved that the optimal incubation temperature for *Aspergillus terreus* was 30 °C and the enzyme production decreased gradually with additional rise in incubation temperature. Similar findings were observed in *Emericella nidulans* (Jayaramu et al. 2010), *Bacillus aryabhattai* (Singh and Srivastava 2012), and *Fusarium equiseti* (EL-Gendy et al. 2021) studies suggesting that the decline in enzyme production was caused by improper enzyme molecule conformation brought on by the denaturation of mesophilic enzymes at higher temperatures.

Among the most important optimization parameters, agitation and aeration rates were regarded as most critical for microorganisms in determining process productivity. Agitation rates improved the mass transfer of oxygen from gas bubbles in the environment and could have an effect on the dissolved oxygen of the medium (Momeni 2015) and the availability of the nutrients in the medium. Results showed that the escalation in the agitation rate aided in mixing of the nutrients which boost its absorption by the microorganisms (Momeni 2015; Abdelrazek et al. 2019). However, a decline in the production of the enzyme was observed at higher agitation speeds which could be credited to the increased shearing forces on the bacterial cells. In our study, increasing the agitation speed from 100 to 180 rpm proved to be useful for the growth of microorganisms and enzyme production, however it began to decline gradually at higher rates. These results were similar to those reported on *Candida utilis* (Momeni 2015), *Aspergillus terreus* (Gurunathan and Sahadevan 2011) and *Bacillus licheniformis* (Abdelrazek et al. 2019).

Concerning aeration effect, the results showed that the optimum production of l-ASNase was observed when using a flask volume 250 ml. Although vigorous aeration aided in gaining large amounts of biomass, nevertheless it reduced the enzyme yield. This might be caused by superficial denaturation of the enzyme protein, like that detected in guinea pig serum l-ASNase or due to the enzyme synthesis inhibition with oxygen as repressor (Mikucki et al. 1977).

It is well known that L-ASNase is a very effective anti-leukemic agent and for most patients it is usually administered once every two weeks (Graham 2003; Oza et al. 2010; Ali et al. 2018). The relative selectivity with regard to metabolism of malignant tumor cell forces us to look for novel l-ASNase compared to existing enzymes (Ali et al. 2018). On investigating the in vitro antileukemic activity of l-ASNase on both malignant and normal cells, results revealed that the carcinoma cell death percentage reached almost 84% upon treatment with high concentrations of the enzyme achieving an IC_50_ of 3.66 µg/ml. Meanwhile, the normal cell death percentage upon treatment with high enzyme concentrations did not exceed 24% with IC_50_ value greater than 300 µg/ml. These results came in agreement with several previous studies indicating that l-ASNase enzyme obtained from different *Fusarium spp*. showed selective toxicity against various malignant cells while having low toxicity on normal cells. In 2018, a study reported that cell death percentage of carcinoma cell lines was reached between 70 and 80% after treatment with the crude l-ASNase of either *Aspergillus sydowii* or *Fusarium oxysporum*, respectively (Ali et al. 2018). Purified l-ASNase from *Fusarium equiseti* was isolated in a different study, and demonstrated promising anti-proliferative activity towards different malignant cell lines including: cervical epitheloid carcinoma (Hela), epidermoid larynx carcinoma (Hep-2), hepatocellular carcinoma (HepG-2), colorectal carcinoma (HCT-116), in addition to breast adenocarcinoma (MCF-7), with IC_50_ values of 2.0, 5.0, 12.40, 8.26 and 22.8 µg/mL, respectively. The enzyme exhibited less cytotoxicity toward normal cells (WI-38) and greater activity, selectivity, and anti-proliferative activity in a dose-dependent manner towards malignant cells (EL-Gendy et al. 2021). A recent study investigating the cytotoxic influence of l-ASNase from *Fusarium* sp. against RAW2674 leukemic cell lines also revealed antileukemic activity with IC_50_ of 50.1 UmL^−1^ (Al Yousef 2022). The enzyme's selectivity for malignant cells is primarily due to their high reliance on l-ASNase to maintain malignant growth, as it lacks l-asparagine synthetase. Meanwhile, normal cells were not affected as they were able to synthesize l-asparagine with the aid of l-asparagine synthetase which was present in sufficient amounts (Narta et al. 2007; Al Yousef 2022).

In conclusion, endophytic fungus, *Fusarium solani*, obtained from the well known medicinal plant *Hedera helix* L. was revealed to be a successful source for production of l-ASNase. Maximization of enzyme production was achieved under suitable nutritional and environmental conditions. To find its path for use in the realm of medicine, more tests are required to concentrate, purify, lyophilize, and preserve this significant enzyme.

## Data Availability

Please contact author for data request.

## Abbreviations

ALL: Acute lymphoblastic leukemia
WBCs: White blood cells
L-ASNase: L-asparginase
PDA: Potato Dextrose Agar
ITS: Internal transcribed spacer
PCR: Polymerase chain reaction
NCBI: National center for biotechnology information
BLAST: Basic local alignment search tool
TCA: Tri-chloroacetic acid
BDB: Potato dextrose broth
HSF: Human skin fibroblast
RPMI: Roswell Park Memorial Institute
DMEM: Dulbecco’s Modified Eagle Medium
WST-1: Water-soluble tetrazolium -1
